# Preclinical characterisation of gallium-68 labeled ferrichrome siderophore stereoisomers for PET imaging applications

**DOI:** 10.1186/s41181-024-00249-z

**Published:** 2024-03-04

**Authors:** Kristyna Krasulova, Barbora Neuzilova, Katerina Dvorakova Bendova, Zbynek Novy, Miroslav Popper, Marian Hajduch, Milos Petrik

**Affiliations:** 1 Institute of Molecular and Translational Medicine, Faculty of Medicine and Dentistry, Palacky University, Hnevotinska 5, 779 00 Olomouc, Czech Republic; 2 Czech Advanced Technology and Research Institute, Palacky University, Krizkovskeho 511/8, 779 00 Olomouc, Czech Republic; 3 University Hospital Olomouc, I.P. Pavlova 6, 779 00 Olomouc, Czech Republic

**Keywords:** Stereoisomers, Siderophore, Imaging, Infection, Positron emission tomography

## Abstract

**Background:**

Siderophores are small iron-binding molecules produced by microorganisms to facilitate iron acquisition from the environment. Radiolabelled siderophores offer a promising solution for infection imaging, as they can specifically target the pathophysiological mechanisms of pathogens. Gallium-68 can replace the iron in siderophores, enabling molecular imaging with positron emission tomography (PET). Stereospecific interactions play a crucial role in the recognition of receptors, transporters, and iron utilisation. Furthermore, these interactions have an impact on the host environment, affecting pharmacokinetics and biodistribution. This study examines the influence of siderophore stereoisomerism on imaging properties, with a focus on ferrirubin (FR) and ferrirhodin (FRH), two *cis–trans* isomeric siderophores of the ferrichrome type.

**Results:**

Tested siderophores were labelled with gallium-68 with high radiochemical purity. The resulting complexes differed in their in vitro characteristics. [^68^Ga]Ga-FRH showed less hydrophilic properties and higher protein binding values than [^68^Ga]Ga-FR. The stability studies confirmed the high radiochemical stability of both [^68^Ga]Ga-siderophores in all examined media. Both siderophores were found to be taken up by *S. aureus, K. pneumoniae* and *P. aeruginosa* with similar efficacy. The biodistribution tested in normal mice showed rapid renal clearance with low blood pool retention and fast clearance from examined organs for [^68^Ga]Ga-FR, whereas [^68^Ga]Ga-FRH showed moderate retention in blood, resulting in slower pharmacokinetics. PET/CT imaging of mice injected with [^68^Ga]Ga-FR and [^68^Ga]Ga-FRH confirmed findings from ex vivo biodistribution studies. In a mouse model of *S. aureus* myositis, both radiolabeled siderophores showed radiotracer accumulation at the site of infection.

**Conclusions:**

The ^68^Ga-complexes of stereoisomers ferrirubin and ferrirhodin revealed different pharmacokinetic profiles. In vitro uptake was not affected by isomerism. Both compounds had uptake with the same bacterial culture with similar efficacy. PET/CT imaging showed that the [^68^Ga]Ga-complexes accumulate at the site of *S. aureus* infection, highlighting the potential of [^68^Ga]Ga-FR as a promising tool for infection imaging. In contrast, retention of the radioactivity in the blood was observed for [^68^Ga]Ga-FRH. In conclusion, the stereoisomerism of potential radiotracers should be considered, as even minor structural differences can influence their pharmacokinetics and, consequently, the results of PET imaging.

**Supplementary Information:**

The online version contains supplementary material available at 10.1186/s41181-024-00249-z.

## Background

Siderophores are low molecular weight compounds synthesised and secreted by fungi, bacteria, and some plants. They form highly stable complexes with iron ions that are recognised by specific membrane transporters and imported into the cell (Hider and Kong 2010). Microorganisms, like all living things, need iron to grow. The acquisition of iron from the environment is essential, and siderophore-mediated iron uptake is an important pathway for its acquisition. Therefore, siderophores play a crucial role in the virulence of many pathogens. (Dale et al. 2004). In recent years, siderophores have become the subject of increasing interest as they show great potential in many biomedical applications. They represent a promising tool for therapeutic applications by coupling with therapeutics using a Trojan horse strategy (Liu et al. 2018; Peukert et al. 2023). They have potential as biomarkers for human infections (Carroll et al. 2016; Skriba et al. 2018; Hoenigl et al. 2019; Dobiáš et al. 2021; Namikawa et al. 2022), and radiolabeled siderophores can be used for in vivo imaging of infections (Petrik et al. 2010, 2018, 2021; Bendova et al. 2023).

Rapid and specific diagnostic methods for infections are needed as current approaches have limitations. Radiolabeled probes, such as siderophores, which are directly linked to the pathophysiological processes of the pathogen, represent a specific tool for infection imaging. The iron in siderophores can be replaced by its isosteric diamagnetic substituent Ga^3+^ without activity loss. Gallium isotopes such as gallium-67 and gallium-68 (^68^Ga) are widely used in nuclear medicine for diagnostic imaging applications. ^68^Ga is a positron emitter with a half-life of 68 min that can be easily obtained from a ^68^Ge/^68^Ga generator. As such, it is conveniently used for molecular imaging with positron emission tomography (PET). Siderophores with iron replaced by gallium-68 seem to be promising candidates for PET imaging of infections (Petrik et al. 2017, 2020).

**Fig. 1:**
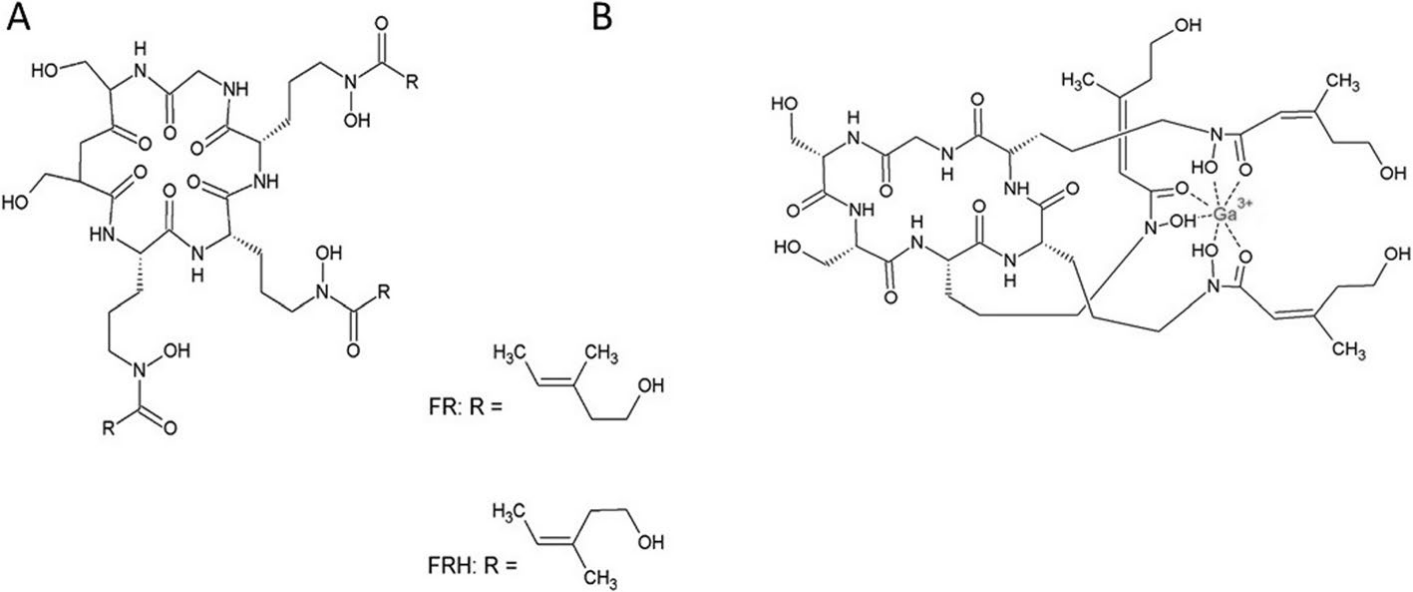
**a** The chemical structure of FR and FRH. **b** The chemical structure of [^68^Ga]Ga-FR and [^68^Ga]Ga-FRH

Ferrirhodin (FRH) and ferrirubin (FR) are microbial ferrichrome-type *cis–trans* isomeric siderophores (Fidelis et al., n.d.). Both are produced by filamentous fungi. Ferrirhodin has been isolated from *Aspergillus versicolor*, *Aspergillus nidulans*, *Aspergillus oryzae*, *Botrytis cinerea* and *Fusarium sacchari*, while ferrirubin is produced by *Aspergillus ochraceus* (Jalal et al. 1984; Huschka et al. 1986). Ferrichromes are cyclic hexapeptides consisting of three N5-acylated N5-hydroxyornithine residues (R4-R6), which provide the hydroxamate groups for iron binding and three additional amino acids (Aguiar et al. 2021). This pair of molecules has the same formula, but the acyl groups of R4-R6 (anhydromevalonic acids) have different orientations in the three-dimensional space. The *cis* stereoisomer, ferrirubin, has the anhydromevalonic acid on the same side of the plane, whereas the *trans* ferrirhodin has the same group on the opposite side (Fig. 1a, b). The *cis/trans* configuration of the anhydromevalonic acid in FR and FRH structures results in a different appearance of residues around the iron-binding site.

Stereospecific interactions among biomolecules are ubiquitous, extending even to fundamental microbial activities such as iron acquisition facilitated by siderophores. When considering siderophores in the context of imaging, two distinct levels of interactions come into play. The first involves the interaction between siderophores and bacteria, while the second applies to the interaction between siderophores and the organism undergoing imaging. It is known that the specific three-dimensional structure of the iron-siderophore complex is responsible for the recognition of receptors, transporters and iron utilisation (Huschka et al. 1986; Winkelmann 2002; Raymond et al. 2015). Stereospecific recognition has been demonstrated with the enantiomeric siderophores pyochelin and enantiopyochelin. Different *Pseudomonas* strains produce both, and they are recognised and transported by their various specific outer membrane transporters: FptA in *P. aeruginosa* and FetA in *P. fluorescens* (Brillet et al. 2011). The situation is different for the *E. coli* siderophore enterobactin and its enantiomer, enantio-enterobactin. Both are bound by the same outer membrane receptor with similar affinity, but there is a difference in uptake and iron release (Raymond et al. 2015). Stereospecific interaction on the level of the host environment involves different pharmacokinetic profiles of stereoisomers. Isomers may bind differently to tissues and blood components such as plasma proteins, blood cells or lipoproteins. This may result in different plasma concentrations, elimination rates, plasma half-lives and biodistribution (Brooks et al. 2011; Coelho et al. 2021).

Here we report on the effect of siderophore stereoisomerism on their imaging properties. We evaluated two isomeric siderophores, ferrirhodin and ferrirubin, radiolabelled with ^68^Ga, to investigate their in vitro properties and in vivo behaviour as well as their potential for molecular imaging of infections by positron emission tomography. To this end, in vitro assays, animal experiments in healthy mice and initial PET imaging in mouse infection model were performed.

## Materials and methods

### Chemicals

All reagents were purchased as reagent grade from commercial sources and used without further purification. HPLC-pure ferrirubin and ferrirhodin were obtained from Biophore Research Products (Tübingen, Germany). ^68^GaCl_3_ was obtained from a ^68^Ge/^68^Ga-generator (Eckert & Ziegler Eurotope GmbH, Berlin, Germany) using a fractionated elution method with 0.1 M HCl (Petrik et al. 2011).

### Radiolabeling

FR and FRH stock solutions were prepared by water dissolving (1 μg/μL). Each substance was radiolabeled as follows: 5 μL of stock solution was mixed with 30 μL of sodium acetate (155 mg/mL in water) and 300 μL of ^68^GaCl_3_ generator eluate (15 − 40 MBq). The reaction mixture was incubated for 5 min at room temperature, and then the pH was adjusted to 6–7 by adding 100 μL of sodium acetate (155 mg/mL). The radiochemical purity of the radiolabeled siderophores was monitored by reversed-phase high-performance liquid chromatography (RP-HPLC) and instant thin-layer chromatography on silica gel-impregnated glass fibres (iTLC-SG). RP-HPLC was performed using the Dionex Ultimate 3000 system (Dionex UltiMate 3000, Thermo Scientific, Waltham, MA, USA) in combination with a radiometric detector (GABI Star, Raytest, Straubenhardt, Germany). A column (Nucleosil 120–5 C18 250 × 40 mm, WATREX, Prague, Czech Republic) with a flow rate of 1 mL/min, oven temperature of 25 °C, and UV detection at 225 and 250 nm was used with acetonitrile (ACN)/0.1% trifluoroacetic acid /H_2_O as mobile phase with the following gradient: 0–2 min-0% ACN; 2–15 min—0–36% ACN; 15–18 min—36–60% ACN; 18–19.5 min—60% ACN; 19.5–20 min—60–0% ACN; 20–24 min—0% ACN.

Silica-gel-impregnated glass microfibre chromatographic papers (Varian, Lake Forest, CA, USA) were used for iTLC-SG analysis. Chromatographic paper strips containing a sample of the [^68^Ga]Ga-FR/FRH complex were developed in a chamber saturated with ammonium acetate (1 M) and methanol 1: 1. After development, the strips were scanned using a radiometric phosphor imager (Cyclone Plus Storage Phosphor System, PerkinElmer, Waltham, MA, USA) and the chromatograms for each strip were evaluated.

### In vitro characterisation

#### Log *P*

The partition coefficient (log *P*) was determined by adding 350 μL of the [^68^Ga]Ga-FR/FRH reaction mixture to 650 μL phosphate-buffered saline (PBS). A 50 μL sample was taken from this dilution and mixed with 450 μL PBS and 500 μL octanol. This solution was vortexed (1500 rpm, 20 min) and then centrifuged (1 min, 15,000*g*) to separate the solvents. A 50 μL sample was taken from the aqueous and organic phases and then measured on a γ-counter (2480 Wizard2 automatic gamma counter; PerkinElmer, Waltham, MA, USA). Log *P* was then calculated from data (mean of n = 6).

#### Protein binding

Plasma protein binding was determined by incubating 50 μL of the [^68^Ga]Ga-FR/FRH reaction mixture with 450 μL human serum (or 450 μL PBS as control) at 37 °C for 30, 60 and 120 min. At each time, 25 μL of the sample was separated by size exclusion chromatography (MicroSpin G-50 columns, Sephadex G-50, GE Healthcare, Buckinghamshire, UK) by centrifugation at 2000*g* for 2 min. Protein binding of [^68^Ga]Ga-FR/FRH was determined by measuring the distribution of activity between column (non-protein bound fraction) and eluate (protein bound fraction) using a γ-counter.

#### In vitro stability tests

Stability tests were performed by preparing five samples: (1) a reaction mixture of the 100 μL [^68^Ga]Ga-RF/FRH and 300 μL human serum; (2) 100 μL [^68^Ga]Ga-FR/FRH and 100 μL FeCl_3_ as a competing cation (0.1 M); (3) 100 μL [^68^Ga]Ga-FR/FRH and 100 μL FeCl_3_ (0.1 mM); (4) 100 μL [^68^Ga]Ga-FR/FRH and 100 μL of diethylenetriaminepentaacetic acid (DTPA, 6 mM) as a competing chelator; and (5) a 100 μL reaction mixture containing only [^68^Ga]Ga-FR/FRH. All samples were incubated at 37 °C for 30, 60 and 120 min. After incubation, acetonitrile was added to human serum samples; samples were centrifuged (15,000 rpm, 3 min), and the supernatant was analysed with RP-HPLC. As described above, other samples were analysed directly by RP-HPLC or iTLC-SG. Incubations were performed in three independent measurements.

### Microbial strains and growth conditions

All microbial strains used in this study were obtained from commercial culture collections. The bacterial strains were cultured on Petri dishes containing Columbia blood agar for 24 h at 37 °C. After culturing on a solid medium, the bacterial mass was transferred to 10 mL of Mueller–Hinton broth (MH) or Minimal salts medium (M9) and shaken at 120 rpm for 24 h at 37 °C. The following strains were tested: *Staphylococcus aureus CCM597, Pseudomonas aeruginosa* ATCC15692, *Klebsiella pneumoniae* NCTC13465, *Escherichia coli* CRC10/CRC/2014 and *Candida albicans* ATCC64550.

### In vitro uptake assays of [^68^Ga]Ga-FR and [^68^Ga]Ga-FRH

For the in vitro uptake assays, [^68^Ga]Ga-FR or [^68^Ga]Ga-FRH (c ∼ 200 nM) was incubated with different microbial strains for 45 min at 37 °C in Eppendorf tubes shaken at 300 rpm. The incubation was terminated by centrifugation at 15,000 rpm for 5 min, after which the supernatant was removed, and the microbial pellet was rinsed with ice-cold Tris buffer (10 mM tris(hydroxymethyl)aminomethane in 0.9% NaCl). The tubes with the microbial pellet were weighed, and the activity was measured by γ-counter. The results were expressed as the percentage of applied dose per gram of microbial culture (% AD/g). The specificity of in vitro uptake of [^68^Ga]Ga-FR and [^68^Ga]Ga-FRH was tested in *S. aureus, P. aeruginosa* and *K. pneumoniae* cultures. The microbial cultures were inhibited by heating at 90 °C for 40 min to prove specific uptake. To determine the uptake of studied siderophores in the presence of an iron-sufficient environment, [^68^Ga]Ga-FR and [^68^Ga]Ga-FRH were pre-incubated for 15 min with iron-siderophore complex (15 mM Fe-desferrioxamine). Then, the samples were handled as described above. To characterise the time–dependence of uptake, [^68^Ga]Ga-FR and [^68^Ga]Ga-FRH were incubated with studied bacterial strains for 10, 20, 30, 45, 60 and 90 min.

### Animal experiments

Animal experiments were performed on female BALB/c mice, 8 to 10 weeks old (Envigo, Horst, The Netherlands). The animals were acclimatised to laboratory conditions for one week before the experiments and housed under standard laboratory conditions on sawdust in individually ventilated cages with free access to food and water. General health and body weight were monitored throughout the experiments. For all in vivo experiments, the number of animals was reduced as much as possible (generally n = 3 per group and time point). Injections and small animal imaging were all performed under 2% isoflurane anaesthesia (FORANE, Abbott Laboratories, Abbott Park, IL, USA) to minimise animal suffering and prevent animal movement. All animal experiments were done following the regulations and guidelines of the Czech Animal Protection Act (No. 246/1992) and with the approval of the Czech Ministry of Education, Youth, and Sports (MSMT-9487/2019- 5 and MSMT-24421/2021-4) and the Institutional Animal Welfare Committee of the Faculty of Medicine and Dentistry of Palacky University in Olomouc.

### In vivo stability tests

Healthy BALB/c mice under 2% isoflurane anaesthesia were retro-orbitally (r.o.) injected with [^68^Ga]Ga-FR or [^68^Ga]Ga-FRH at a dose of 5–10 MBq per animal, and urine or blood was examined. A urine was collected 30 and 90 min after injection. An aliquot of the urine was injected into the RP-HPLC system and analysed. Blood was obtained retro-orbitally 5 min after injection and centrifuged at 5 000 rpm for 10 min to separate the plasma. The plasma was deproteinised by adding acetonitrile, vortexed for 1 min and centrifuged at 15 000 rpm for 5 min. The supernatant was collected for RP-HPLC analysis.

### Ex vivo biodistribution in healthy mice

Biodistribution studies were performed in healthy BALB/c mice. Mice were r.o. injected with [^68^Ga]Ga-FR or [^68^Ga]Ga-FRH (1 − 2 MBq, approximately ∼ 0.5 μg of the siderophore). All mice were sacrificed 30 and 90 min after injection under general anaesthesia by cervical dislocation followed by exsanguination. Blood, spleen, pancreas, stomach, intestine, kidneys, liver, heart, lung, muscle, and bone were collected; then, the organs and tissues were weighed, and radioactivity was measured using the γ-counter. Biodistribution data were calculated as the percentage of injected dose per gram of tissue (% ID/g).

### PET/CT imaging

The experimental animals under isoflurane anaesthesia were injected r.o. with [^68^Ga]Ga-FR or [^68^Ga]Ga-FRH (approximately ∼ 0.5 μg of siderophore) at a dose of 5–8 MBq per animal and placed in the prone position in the Mediso NanoScan PET/CT small animal imaging system (Mediso Medical Imaging Systems, Budapest, Hungary). After the administration of [^68^Ga]Ga-FR or [^68^Ga]Ga-FRH, static imaging was initiated at 30 and 90 min p.i. Dynamic imaging studies were started ∼ 5 min p.i. Single field-of-view PET scans (98.5 mm) were performed, followed by whole-body helical CT scans (50 kVp/980 μA, 720 projections). Image reconstruction was performed using Mediso Tera-Tomo 3D PET iterative reconstruction (Mediso Medical Imaging Systems, Budapest, Hungary). Images were visualised, processed, and quantified using Mediso InterView FUSION (Mediso Medical Imaging Systems, Budapest, Hungary). Final images were normalised to injected activity and animal weight.

### Animal infection model

In vivo uptake of [^68^Ga]Ga-FR and [^68^Ga]Ga-FRH was studied in a murine model of acute myositis in immunosuppressed BALB/c mice. An intraperitoneal injection of cyclophosphamide (Endoxan, Baxter, Prague, Czech Republic) was administered five and one day before the infection. These injections consisted of 150 mg/kg and 100 mg/kg, respectively. On the day of infection, all mice received an intramuscular injection of 50 µl of bacterial culture containing *S. aureus* (with a concentration of 10^8^ CFU/mL) into the muscle of their left hind leg. To assess the specificity of in vivo uptake of [^68^Ga]Ga-FR or [^68^Ga]Ga-FRH, saline and heat-inactivated *S. aureus* culture was injected into the right hind leg muscle of the animal. The microbial infection was allowed to develop for 5 h, and after that, mice were scanned on PET/CT using tested ^68^Ga-siderophores.

### Statistics

All statistical analyses were performed by GraphPad Prism version 8.0 for Windows (GraphPad Software, La Jolla, CA, USA). Data were analysed using the t-test. All present graphs include error bars, which denote the standard deviation. Other data are reported as the mean value ± standard deviation.

## Results

### ^68^Ga labeling and in vitro characterisation of studied siderophores

FR and FRH were radiolabeled with ^68^Ga with a molar activity of up to 8 GBq/µmol. Radiochemical purity was verified by RP-HPLC and iTLC-SG methods described in Materials and methods section. RP-HPLC analysis of [^68^Ga]Ga-FR revealed a principal peak with a retention time of 14.4 min, corresponding to [^68^Ga]Ga-FR and two minor peaks at 12.8 and 13.5 min, possibly indicating other FR isomers or different types of ferrichrome siderophores (Jalal et al. 1984). Nevertheless, upon integration, the radiochemical purity of the main peak exceeded 91%. [^68^Ga]Ga-FRH appeared as a single peak with a retention time of 15.3 min and the radiochemical purity above 99% (see Additional file 1: Figure S1). iTLC-SG analysis confirmed high radiochemical purity for both [^68^Ga]Ga-FR and [^68^Ga]Ga-FRH, which was above 96% for both radiotracers (see Additional file 1: Figure S2).

The resulting complexes differed in their in vitro characteristics (summarised in Table 1). [^68^Ga]Ga-FRH showed less hydrophilic properties (log *P* = − 1.91 compared to − 2.72, respectively) and higher plasma protein binding (~ 50% vs. 6% after 120 min incubation) than [^68^Ga]Ga-FR. The stability studies revealed high in vitro stability of both ^68^Ga-siderophores in examined media, i.e., human serum, 6 mM DTPA, PBS and 0.1 mM FeCl_3_. In the presence of 0.1 M FeCl_3_, ^68^Ga-complexes were unstable due to the high concentration of Fe^3+^ replacing Ga^3+^ in the bond.

**Table 1:** In vitro characterisation results of [^68^Ga]Ga-FR and [^68^Ga]Ga-FRH complexes. Log *P*, protein binding (expressed as a percentage of plasma protein-bound activity of the total activity used) and stability in human serum, 6 mM DTPA, PBS, 0.1 M FeCl_3_ and 0.1 mM FeCl_3_

	[^68^Ga]Ga-FR			[^68^Ga]Ga-FRH				
Log *P* (n = 3)	− 2.72 ± 0.12			− 1.91 ± 0.11				
Incubation time (min)	30	60	120	30		60		120
Protein binding (%) (n = 2)	5.78 ± 2.26	6.55 ± 1.97	6.7 ± 2.52	50.77 ± 1.53		47.24 ± 3.83		51.11 ± 2.90
Stability in human serum (%) (n = 3)	94.26 ± 4.86	94.48 ± 4.61	90.35 ± 8.46	98.55 ± 2.47		99.08 ± 1.54		98.88 ± 1.89
Stability in 0.1 M FeCl_3_ (%) (n = 3)	5.67 ± 2.91	4.13 ± 2.49	4.90 ± 1.81	3.93 ± 3.85		0.40 ± 0.52		0.80 ± 0.46
Stability in 0.1 mM FeCl_3_ (%) (n = 3)	95.97 ± 0.85	96.63 ± 0.25	94.70 ± 0.62	98.75 ± 1.18		98.77 ± 1.20		99.47 ± 1.89
Stability in 6 mM DTPA (%) (n = 3)	94.57 ± 3.04	94.37 ± 1.88	93.77 ± 1.62	98.00 ± 1.05		98.63 ± 1.80		97.98 ± 2.53
Stability in PBS (%)(n = 3)	94.30 ± 2.91	95.93 ± 1.50	95.40 ± 0.68	98.34 ± 1.76		99.33 ± 0.56		99.41 ± 1.02

### In vitro uptake assays

**Fig. 2:**
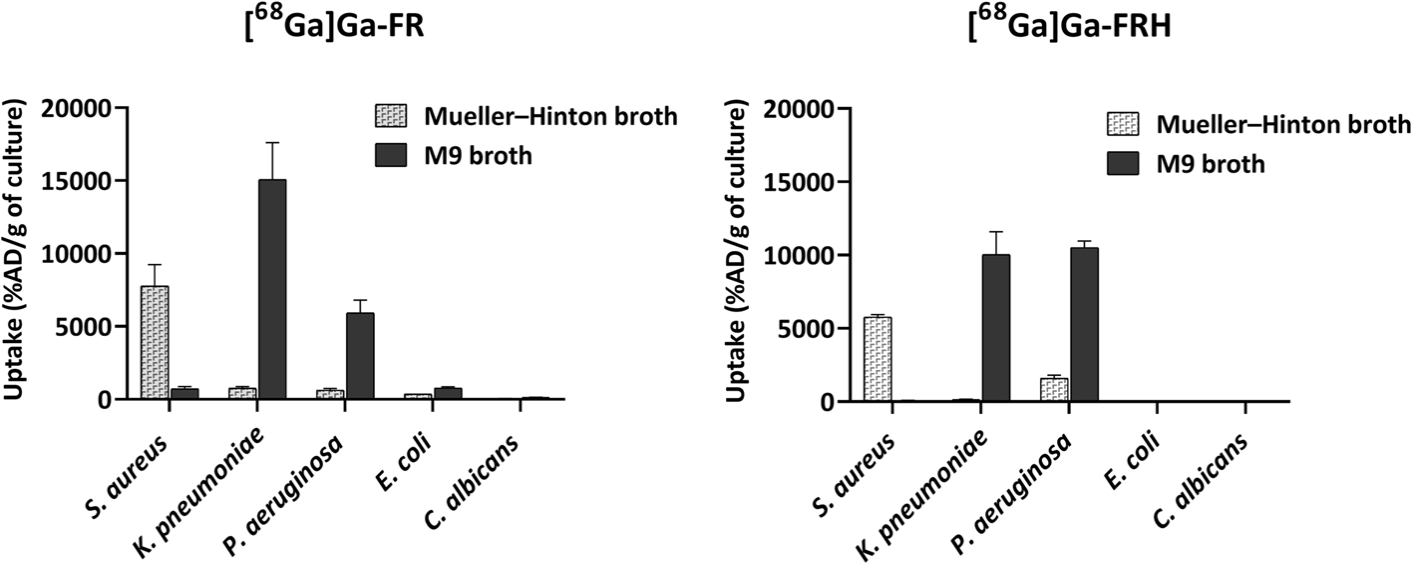
In vitro uptake of [^68^Ga]Ga-FR and [^68^Ga]Ga-FRH in different microbial cultures 45 min after incubation- S. aureus *CCM597,* K. *pneumoniae* NCTC13465, *P. aeruginosa* ATCC15692, *E. coli* CRC10/CRC/2014 and *C. albicans* ATCC64550

**Fig. 3:**
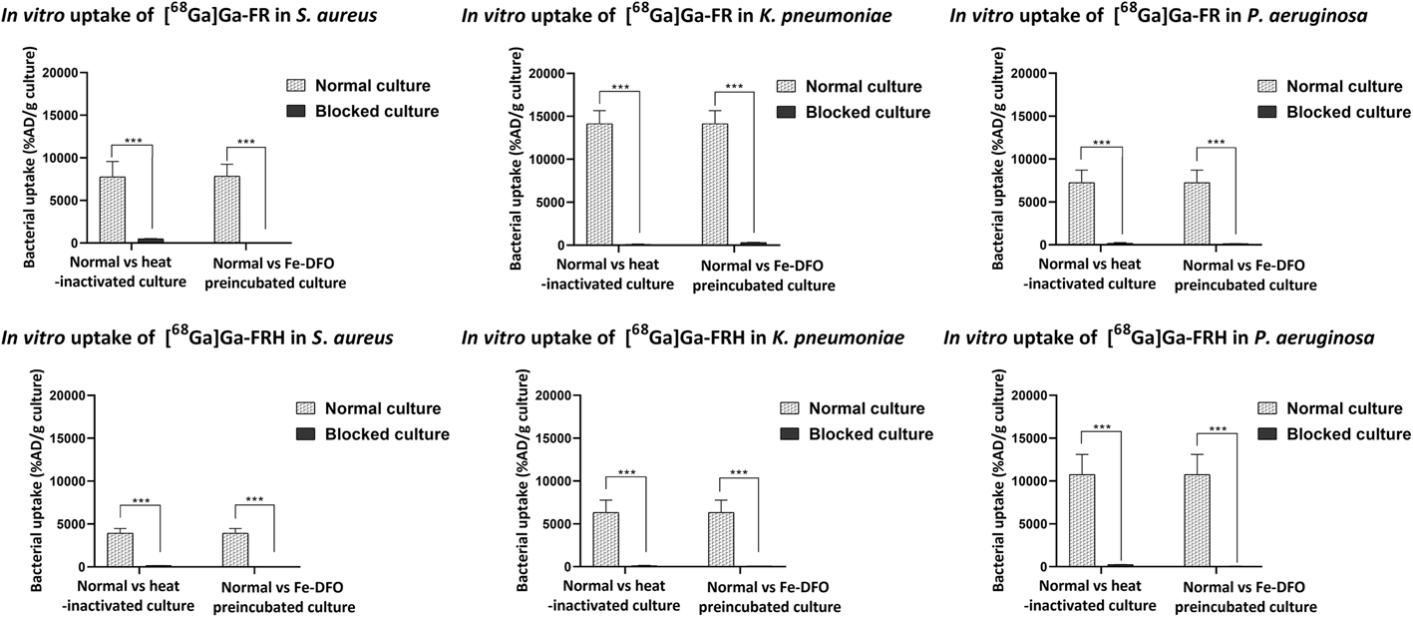
In vitro uptake of [^68^Ga]Ga-FR and [^68^Ga]Ga-FRH after 45 min of incubation in a normal culture of *S. aureus*, *K. pneumoniae* and *P. aeruginosa* compared to a heat-inactivated culture (90 °C, 20 min) and a culture pre-incubated with an excess of Fe-DFO; ***P < 0.01

**Fig. 4:**
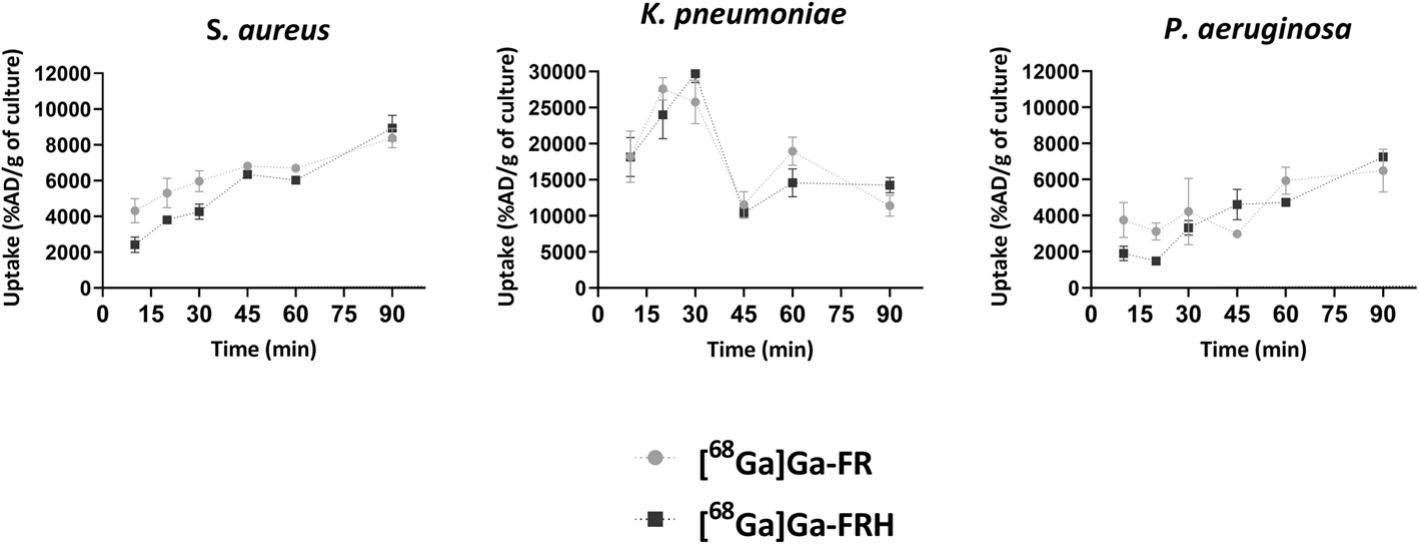
Time-dependent characterisation of in vitro uptake of [^68^Ga]Ga-FR and [^68^Ga]Ga-FRH in selected microbial cultures

Uptake of [^68^Ga]Ga-FR and [^68^Ga]Ga-FRH was tested in five different microbial cultures. Both radiolabeled isomers displayed uptake in the same microbial cultures with slightly different efficacy. The highest uptake was present in *S. aureus, P. aeruginosa and K. pneumoniae*, while *E. coli* and *C. albicans* showed negligible uptake (Fig. 2). Heat-inactivated bacterial cultures (*S. aureus*, *K. pneumoniae.*, *P. aeruginosa*) displayed significantly diminished uptake of both tested siderophores (Fig. 3). Microbial cultures were 15 min pre-incubated at 37 °C with Fe-desferrioxamine (Fe-DFO) before the addition of [^68^Ga]Ga-FR and [^68^Ga]Ga-FRH to the reaction. This pre-incubation significantly reduced the uptake of siderophores by the bacteria (Fig. 3). In the time-dependence experiment, the uptake was seen up to 90 min after incubation without saturation in *S. aureus* and *P. aeruginosa*. Uptake by *K. pneumoniae* showed a maximum after 30 min and then started to decrease (Fig. 4).

### In vivo stability in mice

Stability in urine and blood was tested in healthy BALB/c mice. Both ^68^Ga-complexes were highly stable in vivo (Additional file 1: Figure S3)*.* Even 90 min post-administration, the ratio of [^68^Ga]Ga-FR and [^68^Ga]Ga-FRH complexes to free gallium-68 in urine remained consistently above 89%. Stability in blood was measured 5 min after administration and exceeded 99% for both ^68^Ga-complexes (Table 2).

**Table 2:** In vivo stability of [^68^Ga]Ga-FR and [^68^Ga]Ga-FRH in mice

	Incubation time (min)	Stability in urine (n = 3) (%)	Incubation time (min)	Stability in the blood (n = 3) (%)
[^68^Ga]Ga-FR	0	91.61 ± 0.51	5	78.07 ± 7.46
	30	90.69 ± 1.49		
	90	80.58 ± 4.16		
[^68^Ga]Ga-FRH	0	99.50 ± 0.38	5	99.90 ± 0.05
	30	98.47 ± 0.47		
	90	98.22 ± 1.15		

### Ex vivo biodistribution in mice

**Fig. 5:**
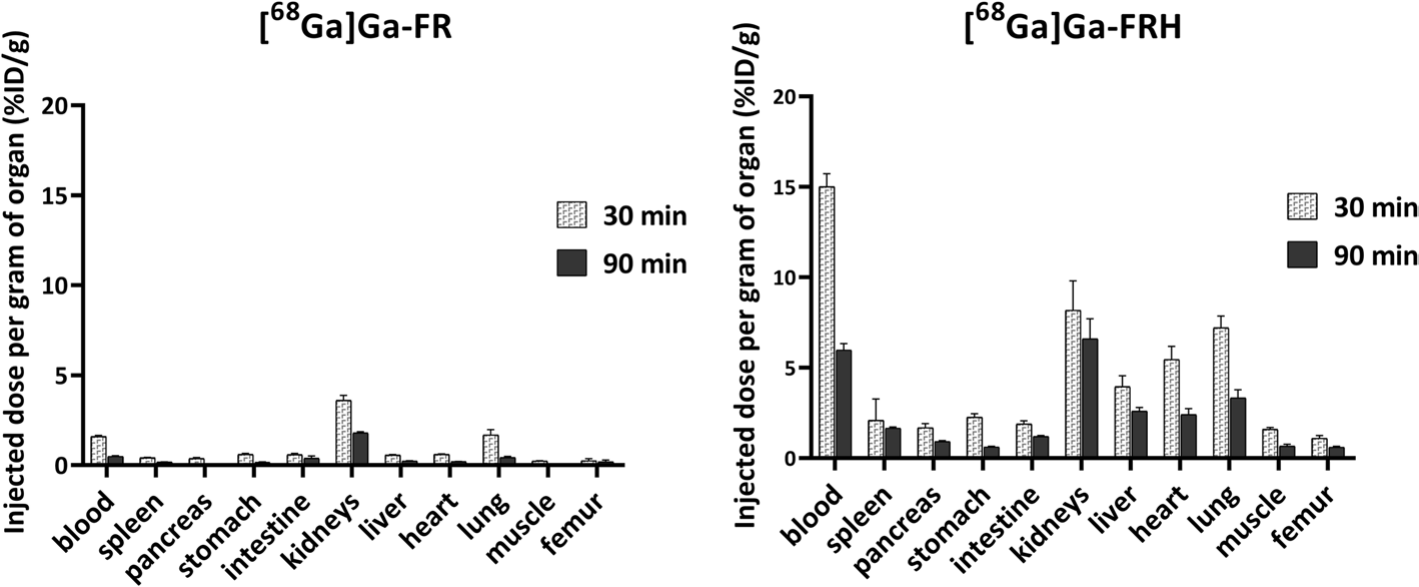
Ex vivo biodistribution of [^68^Ga]Ga-FR and [^68^Ga]Ga-FRH in healthy BALB/c mice 30- and 90- min p.i

The biodistribution of [^68^Ga]Ga-FR tested in healthy BALB/c mice exhibited rapid renal clearance with low blood pool retention (1.59 ± 0.06%ID/g 30 min, 0.49 ± 0.03%ID/g 90 min) and fast clearance from examined organs. Whereas [^68^Ga]Ga-FRH showed moderate retention in blood (15.01 ± 0.71%ID/g 30 min, 5.98 ± 0.35%ID/g 90 min) with slightly higher accumulation in perfused organs. The highest activity concentration in the studied organs at late time points was found for kidneys (6.60 ± 1.11%ID/g 90 min) and lungs (3.34 ± 0.45%ID/g 90 min). The ex vivo biodistribution results are summarised in Fig. 5.

### PET/CT imaging

**Fig. 6:**
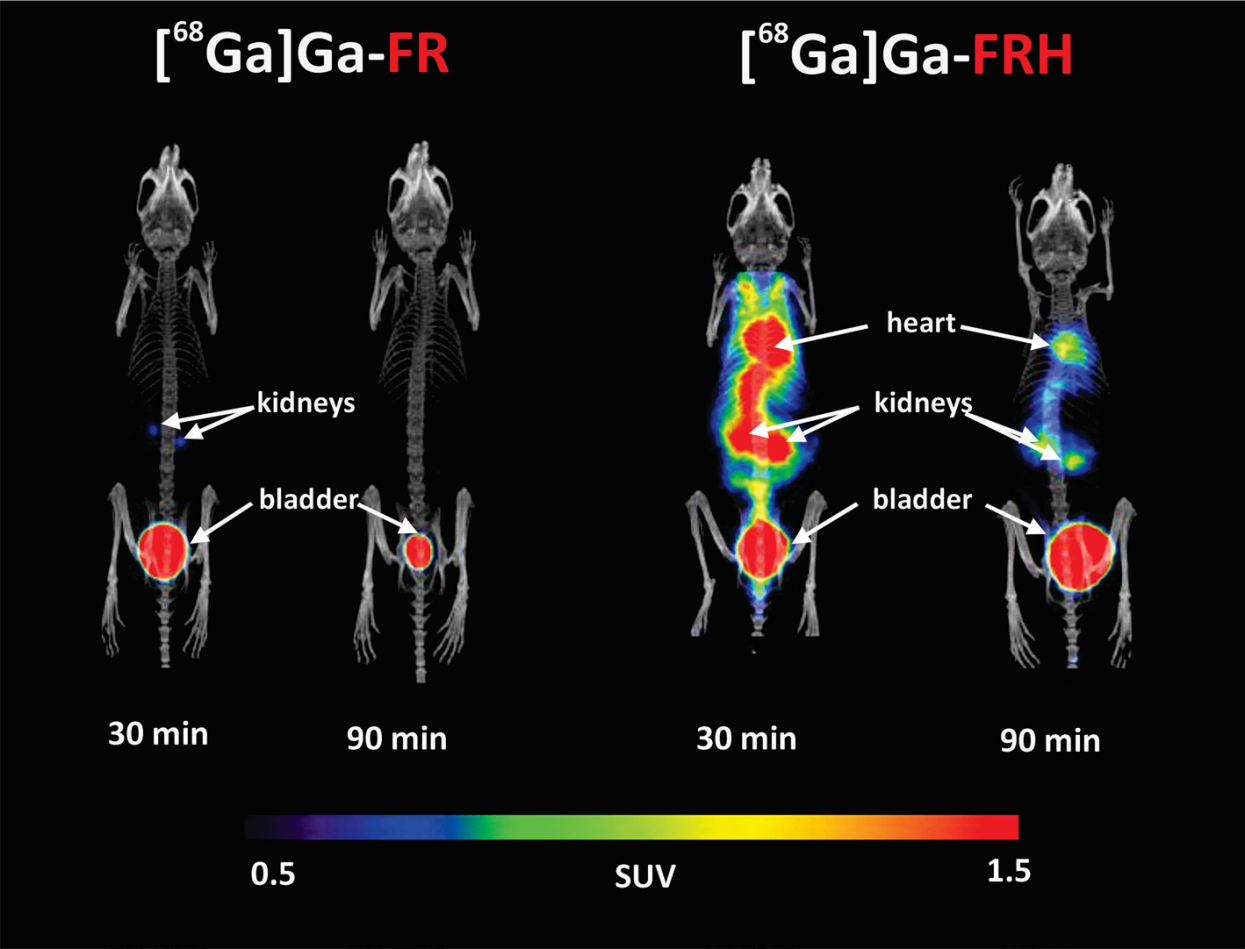
Maximum intensity projection (MIP) PET/CT images of in vivo [^68^Ga]Ga-FR and [^68^Ga]Ga-FRH biodistribution in healthy mice 30 and 90 min after injection of [^68^Ga]Ga-FR/FRH

**Fig. 7:**
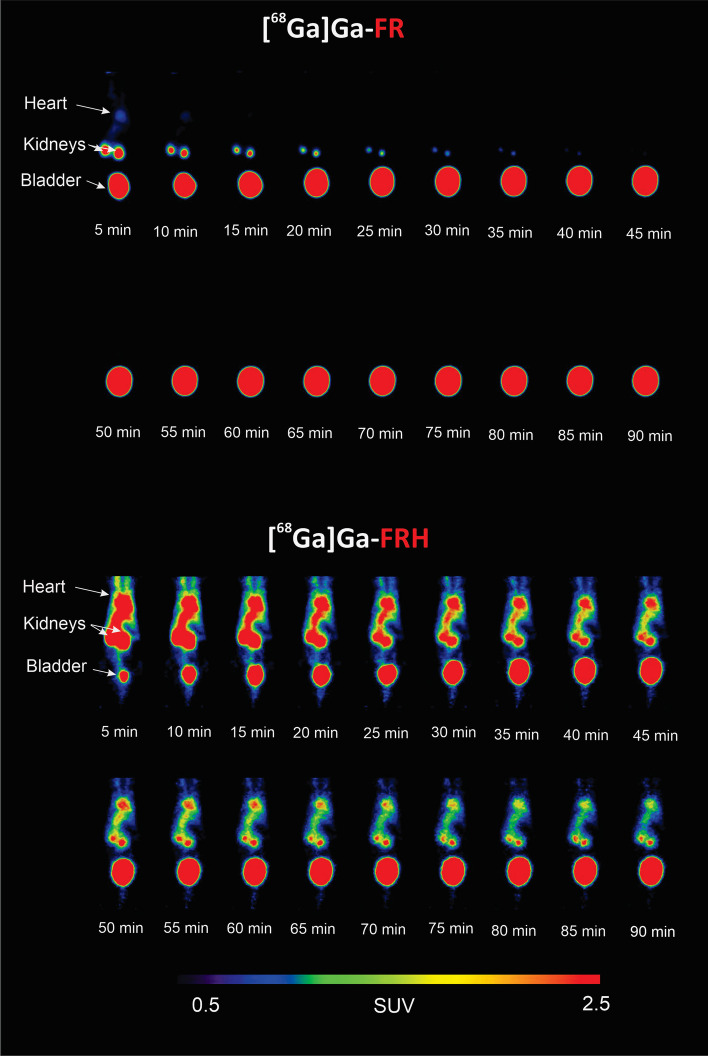
MIP images of PET dynamic in vivo study of [^68^Ga]Ga-FR and [^68^Ga]Ga-FRH biodistribution in healthy mouse up to 90 min after administration

**Fig. 8:**
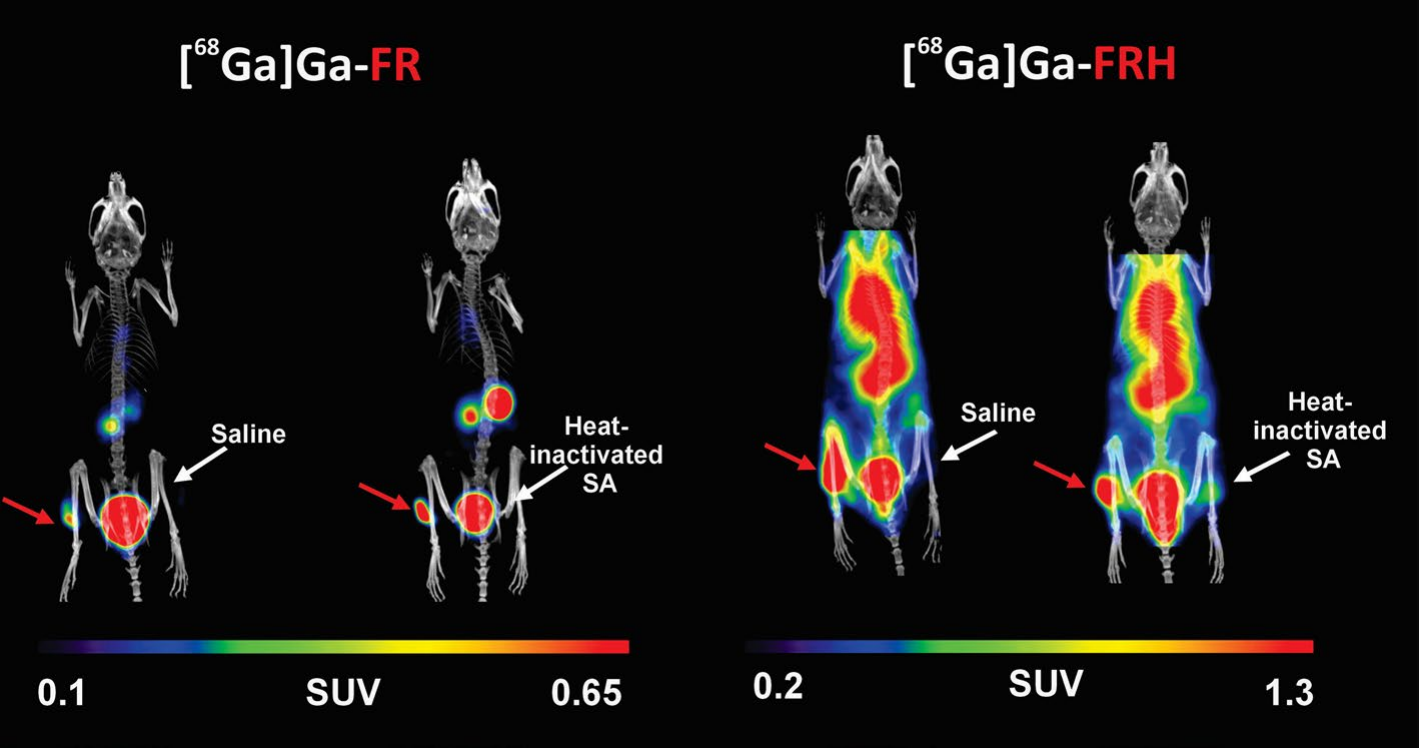
MIP PET/CT images of a mouse model of *S. aureus* myositis in the left hind leg (red arrow) using [^68^Ga]Ga-FR and [^68^Ga]Ga-FRH, while the right hind leg received a saline injection or heat-inactivated *S. aureus* (SA) culture. These MIP images were obtained 5 h after infection and 45 min after radiocomplex administration

PET/CT imaging of mice injected with [^68^Ga]Ga-FR or with [^68^Ga]Ga-FRH confirmed the data from ex vivo biodistribution studies. [^68^Ga]Ga-FR was rapidly cleared from the bloodstream via renal excretion. Also, PET imaging data of [^68^Ga]Ga-FRH agreed with ex vivo biodistribution data and showed radioactivity retention in the bloodstream and several organs (Figs. 6 and 7). In the mouse model of *S. aureus* myositis, the infected left hind leg showed a significant gallium-68 accumulation after [^68^Ga]Ga-FR injection. A similar signal accumulation at the site of infection was obtained using [^68^Ga]Ga-FRH. However, the PET signal was also present in other organs, making this radiotracer unsuitable for infection imaging (Fig. 8). The right hind legs of infected mice injected with saline or heat-inactivated bacteria showed no PET signal for [^68^Ga]Ga-FR or significantly reduced signal for [^68^Ga]Ga-FRH.

## Discussion

Molecular imaging has a great potential for detecting and monitoring human infections. Traditional diagnostic techniques such as microbiological culture, staining, histopathology, serology or molecular methods are often slow, unspecific, invasive and have low sensitivity or accuracy. Infection imaging represents a new approach that can improve the speed and precision of infection diagnosis and, when combined with traditional techniques, provide a more comprehensive assessment of infection (Ordonez and Jain 2018; Kleynhans et al. 2023). Radiolabelled siderophores are one of a promising group of radiotracers under preclinical development that can perform pathogen-specific imaging of infection. Siderophore ability to chelate gallium-68 makes them suitable candidates for PET applications (Petrik et al. 2020). The “ideal” radiotracer criteria are strict, and the pharmacokinetic profile is very critical. Factors such as specificity for receptors, enzymes or transporters, elimination routes, binding to plasma proteins or chemical properties influence the ability of a radiopharmaceutical to stand out. Chirality plays a pivotal role in physiological processes within biological systems, and stereospecific interactions affect a whole range of properties. The presented work focuses on the characterisation of siderophore stereoisomers for gallium-68 labeling, focusing on their application in PET imaging, which has promising prospects especially in infection imaging.

Ferrirhodin and ferrirubin are fungal siderophores with the same structural formula but different 3D orientations of their acyl groups. We have successfully radiolabeled both FR and FRH with gallium-68 with high radiochemical purity. The complexes formed remained stable in human serum, even when exposed to the competing chelating agent DTPA and at iron concentrations greater than 10,000-fold. These results represent suitable properties for a radiotracer (H. Brooks et al. 2011; Ordonez and Jain 2018). The *cis* configuration of [^68^Ga]Ga-FR resulted in more favourable hydrophilic properties and human plasma protein binding values than [^68^Ga]Ga-FRH. Such properties ensure rapid diffusion from the bloodstream into infected tissues, fast clearance from non-target tissues, and elimination via renal excretion (Petrik et al. 2020). [^68^Ga]Ga-FRH shows higher hydrophobicity and high plasma protein binding, thus making it suboptimal for in vivo PET imaging. Such characteristics have previously been described for some other radiolabeled ferrichrome siderophores, ferricrocin and ferrichrome. These were also unstable in human serum and in the presence of DTPA (Petrik et al. 2012). These results show that not all ^68^Ga-siderophores fulfil the conditions for their potential use in nuclear medicine.

Stereospecific interactions between biomolecules are ubiquitous, extending even to fundamental microbial activities such as iron acquisition facilitated by siderophores. Previous studies have documented stereoisomerism in bacterial processes related to iron recognition, uptake, and utilisation involving siderophores such as parabactin, rhodotorulic acid, rhizoferrin, ferrichrome, enterobactin, pyochelin, triscatechol (Winkelmann 2002; Brillet et al. 2011; Raymond et al. 2015; Stow et al. 2021). In our study, rapid in vitro siderophore uptake was observed in *S. aureus*, *K. pneumoniae* and *P. aeruginosa* cultures for both [^68^Ga]Ga-FR and [^68^Ga]Ga-FRH with similar potency. Uptake could be inhibited by using heat-inactivated bacteria or by incubation in an iron-rich medium. *S. aureus* has previously been shown to use siderophores produced by other microorganisms, giving it an advantage over other bacterial communities. It does not produce ferrichrome-type siderophores but can use ferrichrome and, as we have shown, ferrirubin and ferrirhodine (Sebulsky et al. 2003; Conroy et al. 2019). Pathogenic species of *K. pneumoniae* have ten iron uptake systems that can synthesise different types of siderophores, mainly enterobactin, but also ferrichrome, salmochelin, yersiniabactin and even others (Elhaki et al. 2020). The results of this study may indicate that *K. pneumoniae* uses other iron uptake systems, including ferrirubin and ferrirhodin. Hannauer et al. have shown that the inner membrane permease FiuB is involved in ferrichrome uptake by PA (Hannauer et al. 2010). We can assume that the same mechanism is involved in the uptake of ferrirubin and ferrirhodin. The uptake of both ferrichrome siderophores has been described for the first time. According to our observations, stereoisomerism plays a minor role in the microbial recognition of FR and FRH, which explains their similar efficacy in their uptake.

Siderophores, in which gallium-68 replaces iron, have been presented as potentially valuable tool for infection imaging. [^68^Ga]Ga- triacetylfusarinine C for imaging *Aspergillus fumigatus* infection, [^68^Ga]Ga-pyoverdines for imaging *Pseudomonas aeruginosa* infection, [^68^Ga]Ga -desferrioxamine B for imaging various microbial infections and the recent [^68^Ga]Ga-ornibactin for imaging *Burkholderia multivorans* infection, are a list of siderophores with proven potential in animal models of infection (Petrik et al. 2010, 2018, 2021; Bendova et al. 2023). The high potential of these imaging agents has not yet been fully exploited in clinical applications. However, two clinical trials are currently underway using [68 Ga]Ga-DFO for PET in patients with bacterial infections and for PET imaging of infections in patients with vascular grafts (EudraCT Number: 2020-002868-31; NCT05285072).

In this study, we compared in *vitro* properties and in vivo behaviour of two siderophore isomers as well as their potential for imaging *S. aureus* infection. Both ex vivo biodistribution study and in vivo PET/CT in healthy mice proved that [^68^Ga]Ga-FR has an optimal pharmacokinetic profile contrary to the [^68^Ga]Ga-FRH. The PET signal of [^68^Ga]Ga-FRH at the site of infection caused by *S. aureus* interfered with the strong signal in perfused organs caused by blood retention, making it unsuitable for imaging. It is known from previous studies that enantiomers and optical isomers can behave differently in PET imaging. This was shown, for example, in PET studies of ( +)- and (–)-6-[^18^F]fluoronorepinephrine in the heart or comparative PET studies of [^11^C]D-threomethylphenidate and [^11^C]L-threomethylphenidate as a radiotracers to study dopamine transport. Differences in results may be caused by differences in specificity for enzymes and transporters binding to plasma proteins or other pharmacokinetic factors (Ding and Fowler 2003).

## Conclusion

This study reports that the two siderophores ferrirubin and ferrirhodin can be labeled with gallium-68 with high radiochemical purity and excellent stability. Different stereoisomerisms of these compounds resulted in different pharmacokinetic profiles. We have shown that [^68^Ga]Ga-FRH has a high human plasma protein binding, leading to moderate retention in blood. On the other hand, [^68^Ga]Ga-FR has optimal properties for PET imaging. *S. aureus, K. pneumoniae and P. aeruginosa* could uptake both radiocomplexes in vitro. *S. aureus* myositis in mice was used to demonstrate the ability of [^68^Ga]Ga-FR for PET infection imaging. On the contrary, [^68^Ga]Ga-FRH PET scans showed an interference of the infection lesion signal with a high radioactive signal from the blood. We can assume that [^68^Ga]Ga-FRH could also be used for infection imaging if a longer time after injection is used, but this is contradicted by the short half-life of Ga-68. In conclusion, we confirmed here that it is important to consider the stereoisomerism of potential radiotracers. Even small structural variations can affect their pharmacokinetics and, thus, results of the PET imaging.

### Supplementary Information

**Additional file 1**. Supporting Information is provided in addition to data presented in the main manuscript, including representative radiochromatograms regarding quality control and in vivo stability of tested compounds.

## Data Availability

The datasets used and/or analysed during the current study are available from the corresponding authors on reasonable request.

## Abbreviations

^68^Ga: Gallium-68
ACN: Acetonitrile
CT: Computed tomography
DTPA: Diethylenetriaminepentaacetic acid
Fe-DFO: Fe-desferrioxamine
FR: Ferrirubin
FRH: Ferrirhodin
iTLC-SG: Instant thin-layer chromatography on silica gel-impregnated glass fibres
log *P*: The partition coefficient
M9: Minimal salts medium
MIP: Maximum intensity projection
MH: Mueller–Hinton broth
PBS: Phosphate-buffered saline
PET: Positron emission tomography
R.o.: Retro-orbitally
RP-HPLC: Reversed-phase high-performance liquid chromatography
Tris: Tris(hydroxymethyl)aminomethane
