# Supplementary material for: Preclinical characterisation of gallium-68 labeled ferrichrome siderophore stereoisomers for PET imaging applications
Source: EJNMMI Radiopharm Chem. 2024 Mar 4;9:20. doi: 10.1186/s41181-024-00249-z (PMC10912063; doi:10.1186/s41181-024-00249-z)
Supplement: Supplementary file 1 — Additional file 1. Supporting Information is provided in addition to data presented in the main manuscript, including representative radiochromatograms regarding quality control and in vivo stability of tested compounds. [file 41181_2024_249_MOESM1_ESM.docx]

**Supporting information**

**Preclinical characterisation of gallium-68 labeled ferrichrome siderophore stereoisomers for PET imaging applications.**

**Kristyna Krasulova^1^, Barbora Neuzilova^1^, Katerina Dvorakova Bendova^1^, Zbynek Novy^1,2^, Miroslav Popper^1^, Marian Hajduch^1,2,3^ and Milos Petrik^1,2^**

^1^Institute of Molecular and Translational Medicine, Faculty of Medicine and Dentistry, Palacky University, Hnevotinska 5, Olomouc, 779 00, Czech Republic

^2^Czech Advanced Technology and Research Institute, Palacky University, Krizkovskeho 511/8, Olomouc, 779 00, Czech Republic

^3^University Hospital Olomouc, I.P. Pavlova 6, Olomouc, 779 00, Czech Republic.

**Corresponding authors:**

Kristyna Krasulova

Institute of Molecular and Translational Medicine, Hnevotinska 5, CZ-77900 Olomouc, Czech Republic

Tel: +420585632138; Fax: +420585632180; Email: [kristyna.krasulova@upol.cz](mailto:milos.petrik@upol.cz)

https://orcid.org/0000-0002-8303-6847

Milos Petrik

Institute of Molecular and Translational Medicine, Hnevotinska 5, CZ-77900 Olomouc, Czech Republic

Tel: +420585632126; Fax: +420585632180; Email: [milos.petrik@upol.cz](mailto:milos.petrik@upol.cz)

https://orcid.org/0000-0003-1334-5916

**Content**

[1. Figure S1: ^68^Ga-labeling of [^68^Ga]Ga-FR and [^68^Ga]Ga-FRH S3](#_Toc159313016)

[2. Figure S2: Quality control of [^68^Ga]Ga-FR and [^68^Ga]Ga-FRH on radio-iTLC-SG S4](#_Toc159313017)

[3. Figure S3: *In vivo* stability tests S5](#_Toc159313018)

# **Figure S1: ^68^Ga-labeling of [^68^Ga]Ga-FR and [^68^Ga]Ga-FRH**


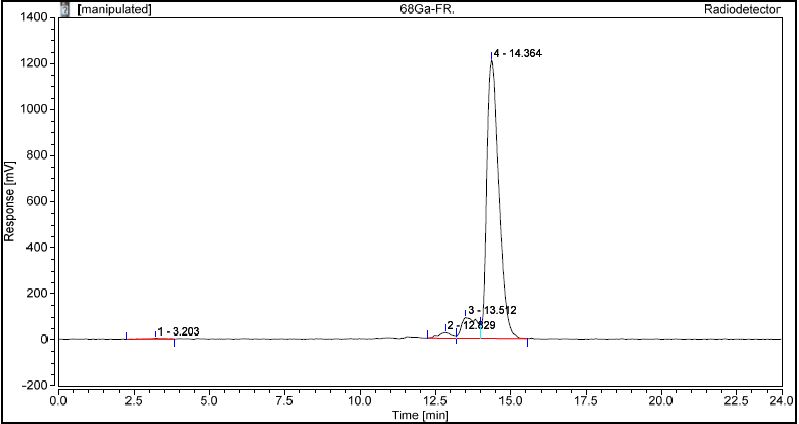


Figure S1A: Example of radiochromatogram of [^68^Ga]Ga-FR.


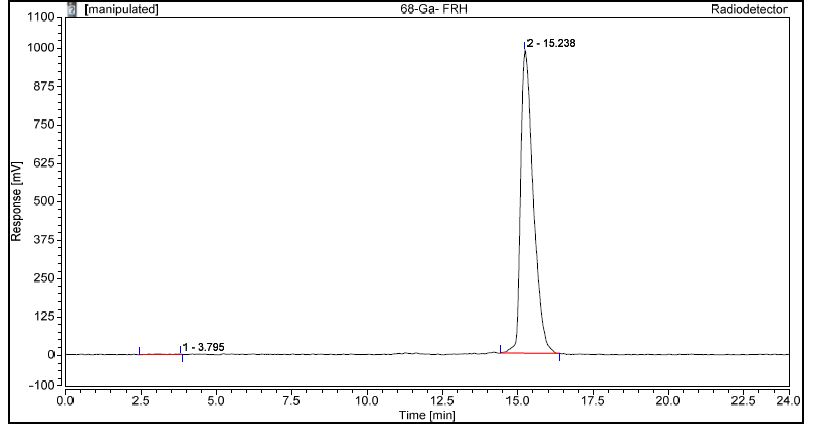


Figure S1B: Example of radiochromatogram of [^68^Ga]Ga-FRH.

#
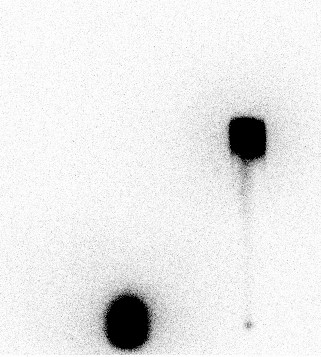
**Figure S2:** **Quality control of [^68^Ga]Ga-FR and [^68^Ga]Ga-FRH on radio-iTLC-SG**

^68^GaCl_3_ generator eluate [^68^Ga]Ga-FR


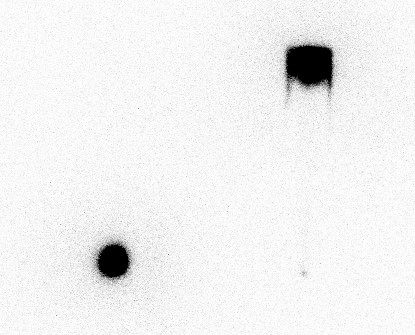
Figure S2A: Radiochromatograms of ^68^GaCl_3_ generator eluate and [^68^Ga]Ga-FR in ammonium acetate (1M) and methanol (1:1).

^68^GaCl_3_ generator eluate [^68^Ga]Ga-FRH

Figure S2B: Radiochromatograms of ^68^GaCl_3_ generator eluate and [^68^Ga]Ga-FR in ammonium acetate (1M) and methanol (1:1).

# **3. Figure S3: *In vivo* stability tests**


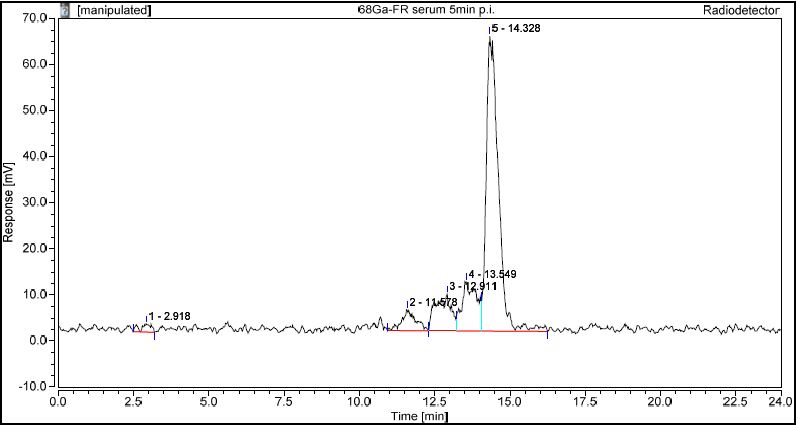


Figure S3A: Radiochromatogram of [^68^Ga]Ga-FR stability in blood, 5 min after injection.


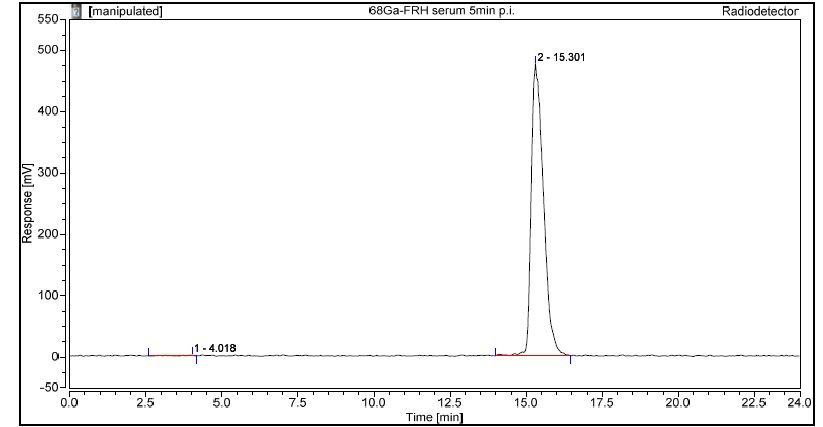


Figure S3B: Radiochromatogram of [^68^Ga]Ga-FRH stability in blood, 5 min after injection.


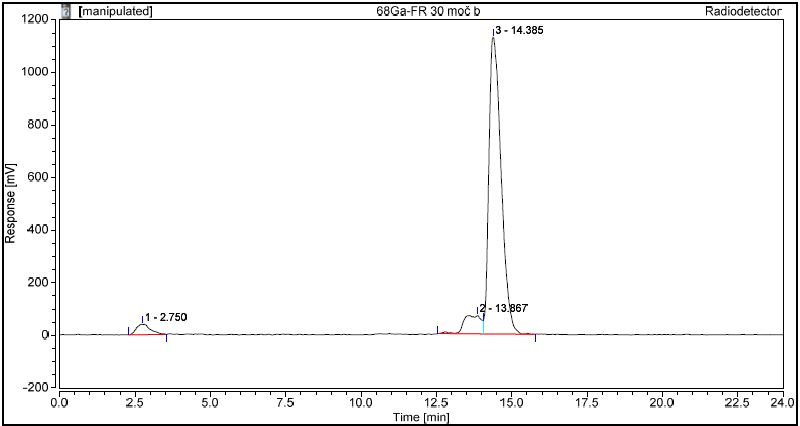


Figure S3C: Radiochromatogram of [^68^Ga]Ga-FR stability in urine, 30 min after injection.


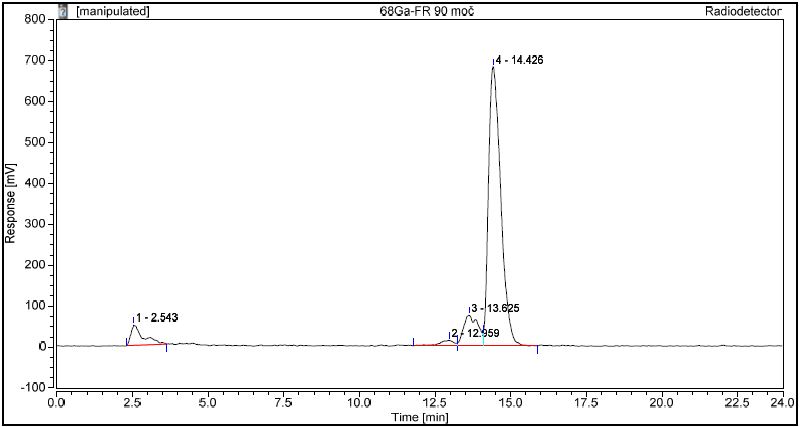


Figure S3D: Radiochromatogram of [^68^Ga]Ga-FR stability in urine, 90 min after injection.


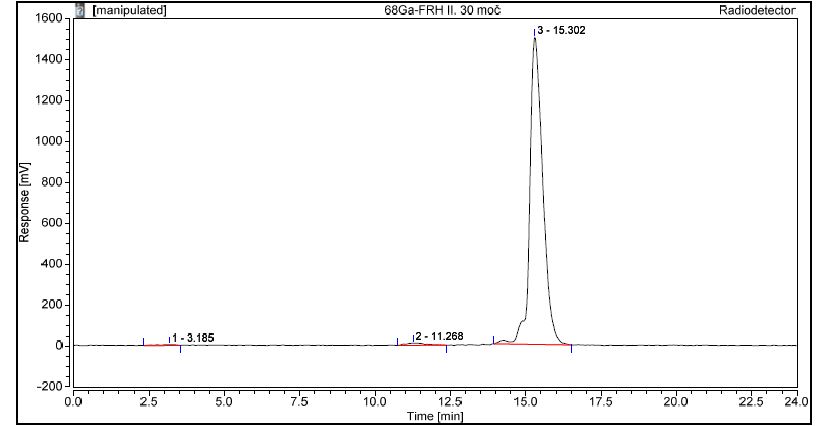


Figure S3E: Radiochromatogram of [^68^Ga]Ga-FRH stability in urine, 30 min after injection.


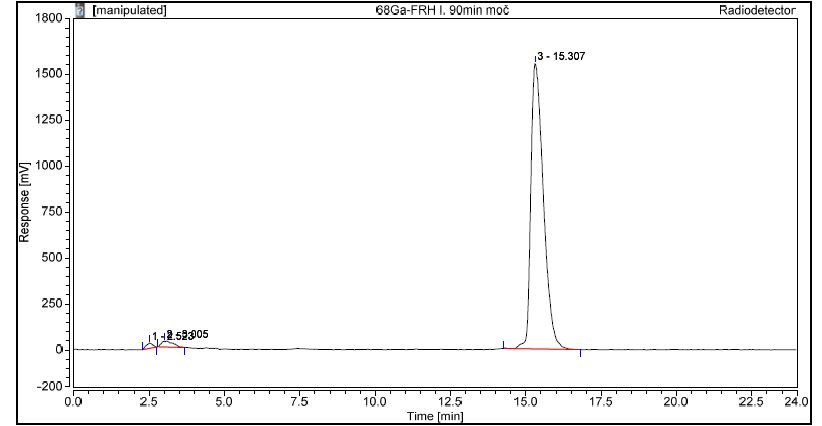


Figure S3F: Radiochromatogram of [^68^Ga]Ga-FRH stability in urine, 90 min after injection.
